# Knowledge and Awareness of Food Safety, Foodborne Diseases, and Microbial Hazards Among Consumers of Street‐Vended Foods in Kandahar City, Afghanistan: A Cross‐Sectional Study

**DOI:** 10.1002/hsr2.71633

**Published:** 2025-12-08

**Authors:** Ahmadullah Zahir, Sayeed Hikmatullah Anis, Safiullah Jauhar

**Affiliations:** ^1^ Faculty of Veterinary Sciences, Department of Food Hygiene and Technology Afghanistan National Agricultural Sciences and Technology University Kandahar Afghanistan; ^2^ Faculty of Basic Sciences, Department of Biology Afghanistan National Agriculture Sciences and Technology University Kandahar Afghanistan; ^3^ Department of Food Technology, Faculty of Agriculture Kabul University Kabul Afghanistan

**Keywords:** cross‐contamination, food safety, public health, street‐vended foods

## Abstract

**Background:**

Common foods found in the streets of Kandahar City include flatbreads (naan), fried stuffed flatbreads (bolani), fried meat (kebabs), rice dishes, fried snacks, seasonal fruit juices, and milk and tea sold from carts, small stalls, or open‐air market counters near busy marketplaces and transportation hubs. These items are often at risk of contamination due to the contaminated environment. Unsafe food is linked to over 200 diseases, from diarrhea to various cancers, and can cause permanent disabilities and death. It is estimated that 600 million people worldwide fall ill from eating contaminated food annually, resulting in a global burden and 420,000 early deaths. This study aimed to assess the level of knowledge and awareness about food safety, foodborne illnesses, and microbial threats among consumers of street‐vended foods (SVFs) in Kandahar City.

**Methods:**

A cross‐sectional survey was conducted involving 500 residents of Kandahar City who regularly purchase and consume SVFs.

**Results:**

The average score reflecting food safety knowledge among consumers was 10.44 (SD = 3.75, range: 3–18), signifying a moderate level of understanding. Consumers belonging to the older demographic (36–45 years) exhibited a higher level of knowledge concerning food safety in comparison to their younger counterparts (18–25 years). A significant proportion of respondents (76%) expressed uncertainty regarding food safety matters when purchasing SVFs, with convenience being cited as the primary reason (34.4%) for acquiring these foods. Furthermore, 45% of the respondents acknowledged that SVFs are more economical than those available in supermarkets and restaurants.

**Conclusions:**

Despite the satisfactory levels of food safety knowledge demonstrated by street food consumers, critical areas such as cross‐contamination and food pathogens require heightened focus in educational initiatives. To enhance hygiene and safety standards for street food in Afghanistan, the Ministry of Health and regulatory bodies must devise effective programs and implement sufficient regulatory measures.

## Summary

- A moderate degree of comprehension about food safety, foodborne illnesses, and microbial threats among consumers of street‐vended foods (SVFs) was ascertained.
- A considerable fraction of participants articulated ambivalence regarding food safety issues when procuring SVFs, with convenience being identified as the principal rationale for selecting these food items.
- Nearly half of the participants recognized that ready‐to‐eat SVFs are more cost‐effective than those offered in supermarkets and dining establishments.

## 1 Introduction

Foodborne diseases (FBDs) are a worldwide issue that significantly endangers human health and can undermine a nation's health infrastructure and economic growth [1]. The World Health Organization (WHO) reports that consuming contaminated food leads to approximately 600 million instances of food poisoning and around 420,000 fatalities each year [2]. Based on these figures, 7.69% of the global population suffers from FBDs annually, suggesting that FBDs are responsible for roughly 7.5% of all deaths worldwide. With more than 100 million reported cases each year, the Eastern Mediterranean Region (EMR) is believed to be the third most affected area concerning FBDs [3, 4]. Street‐vended foods (SVFs) refer to a specific category of food that heightens the risk of FBDs. SVFs are characterized by the sale and distribution of food and beverages by “vendors” in public areas such as streets. These convenient and ready‐to‐eat options are commonly accessible across the globe, as they are typically more affordable than meals obtained in restaurants [5]. Around 2.5 billion people globally consume SVFs daily, making up to 40% of the daily diet for urban inhabitants in low‐ and middle‐income countries. Although they are largely unregulated, SVFs can play a significant role in the local economy within these countries [6].

Concerns about the safety and quality of SVFs persist, as they may harbor enteropathogens such as the hepatitis A virus, *Salmonella*, *Bacillus cereus*, and *Escherichia coli* (*E. coli*) [7]. Based on existing data, more than 200 different types of illnesses can be transmitted through food across the general population. FBDs are associated with long‐lasting adverse effects, particularly impacting children, pregnant women, and the elderly. There is a clear connection between food safety and FBDs, making it vital to elevate food safety standards to curb the spread of these diseases [8]. Annually, around 2 million individuals in developing countries succumb to FBDs, and this figure is expected to rise as food safety has become a significant global public health issue [9]. Due to the insufficient enforcement of food safety regulations and the poor personal and food handling practices among food handlers, developing nations do not uphold food safety standards as rigorously as low‐ and middle‐income countries [10]. This situation has led to severe health consequences for individuals and has also resulted in negative economic impacts [11].

Due to the availability and affordability of SVFs, a significant number of people in Afghanistan consume them daily, which raises food safety concerns. Unfortunately, there is a shift in the drinking habits of young adults, leading to a higher risk of FBDs. Reports indicate a yearly increase in FBDs, with diarrhea being the most prevalent, even though the exact data have not been presented. Regrettably, this situation has resulted in over 50% of deaths among children under 5 years old [11]. One major issue is the contamination of food and water with pesticides, dangerous industrial substances, and waste from humans and animals. Research conducted in Afghanistan shows a high prevalence of various infections found in hand swabs, drinking water, and food items. These include enterotoxins from *E. coli*, *Campylobacter*, *Salmonella*, *Shigella*, rotaviruses, adeno, and hepatitis A virus, as well as intestinal helminths and protozoa such as *Entamoeba histolytica* and *Giardia intestinalis*. It is estimated that nearly 90% of the Afghan population is affected by some form of helminth worm infection. Recent studies in Jalalabad City revealed that 34 samples of SVFs were positive for *Shigella* and *Salmonella*. In another study, these same bacteria were found in 25% and 24% of the chicken livers that were tested and sold on the streets [12].

It's important to know that SVFs can harbor antibiotic‐resistant bacteria such as *Salmonella* and *Enterobacteriaceae*, which raises concerns about microbiological dangers and food safety in relation to public health. The environments and situations in which SVFs are produced and consumed can worsen FBDs, food safety issues, and microbiological risks. Factors like food vendors' lack of knowledge regarding proper food handling practices, the use of untreated water sources, and insufficient hygiene and sanitation during preparation and sale can heighten the likelihood of FBDs and microbiological threats. As the population increases, the risks associated with street food rise [13]. Although this topic has not been widely addressed, it is essential to investigate SVF consumers in developing countries such as Afghanistan to enhance awareness and understanding of food safety, FBDs, and microbiological risks. Previous studies have largely focused on food vendors rather than consumers; therefore, it is necessary to conduct an investigation into food safety among SVF consumers in Afghanistan and to propose effective corrective measures. Kandahar is a rapidly growing urban area in Afghanistan that hosts a variety of food industries. As food demand has surged, there has been an uptick in the number of food establishments. The goals of this research are to (1) assess SVFs consumers' knowledge about food safety and identify differences based on sociodemographic factors; (2) examine the variables that influence their understanding of food safety; and (3) evaluate the level of awareness regarding microbial food safety hazards among SVFs consumers at various eateries in Kandahar City.

## 2 Materials and Methods

### 2.1 Study Sites and Period

The current study, carried out in Kandahar City, Afghanistan, between June and July 2024, focuses on consumers of street food in the area. Kandahar City consists of a total of 15 municipal zones, and this research was conducted across all of them. This city is situated in the western part of Afghanistan, encompassing a sub‐region that represents a considerable share of the country's land area and population, covering 273.4 km² of the overall land area and home to 651,500 residents. The geographic features of this area make it vulnerable to various devastating natural events (such as floods, cyclones, and persistent droughts), which have negatively impacted socio‐economic conditions, as well as water, sanitation, and hygiene issues (including FBDs like diarrhea) experienced by communities living in this region of the country.

### 2.2 Sampling Technique and Sample Size

The method used for sampling was an equal probability cluster sampling approach. The size of the sample was calculated using the Schwartz formula:

$$n=\frac{z^{2}\times p\times \left(1-p\right)}{d^{2}}=\frac{{\left(1.96\right)}^{2}\times 0.5\times \left(1-0.5\right)}{{\left(0.05\right)}^{2}}=384,$$

where *n* = required sample size, *Z* = 1.96 for a 95% confidence interval, *d* = 0.05 (margin of error), *p* = 0.5 (prevalence estimate as no prior study found), and *d* = margin of error (precision) 0.05 (5%).

Given the limited research of this nature in Afghanistan, we employed a single population proportion test to ascertain the sample size. For consumers of SVFs, we applied a 50% prevalence rate regarding awareness of microbiological risks and anticipated food safety. The calculations were conducted with a 95% confidence interval (CI), a 5% margin of error, a 10% non‐response rate, and a design effect factor of 1.5. The minimum sample size required was 384. However, due to the data accuracy and saturation, a total of 500 data points were chosen for further analysis. The distribution of subjects across various zones was based on who buys and consumes SVFs, the density of the population, and access to ensure a varied and representative consumer base.

### 2.3 Data Collection Procedures

A team of six researchers trained by the lead investigator engaged in data collection for a study on ready‐to‐eat SVFs in Kandahar City. At various times throughout the day, when consumers typically buy and enjoy these food items, the team randomly visited different locations in the city where SVFs were available, such as streets, parks, playgrounds, and local markets. Participants for the study, all of whom were required to be at least 18 years old, were selected randomly by the research team. However, 50 individuals chose not to participate for a range of reasons. The research team explained the purpose of the study to the participants and obtained their consent prior to carrying out in‐person interviews using a standardized questionnaire. The interviews, which lasted between 15 and 20 min, took place either at the vending locations or in nearby areas. Before beginning data collection, the research protocols were reviewed, and ethical approval was granted by the Department of Food Science and Technology at Afghanistan Agricultural Sciences and Technology University. The questionnaires were crafted with a focus on protecting respondent anonymity by separating personally identifiable information from publicly available data.

### 2.4 Data Collection Tool

The survey used in this research was adapted from a previous study [4], with slight adjustments. It was organized into several categories, including sociodemographic details, knowledge of food safety, insights into purchasing SVFs, awareness of microbial food safety risks, and perceptions of food safety related to SVFs. Topics included in the sociodemographic section encompassed gender, age, occupation, educational background, marital status, household income, residence location, food safety training, and health certifications. A total of eighteen questions with three possible answers, yes, no, and do not know, were utilized to assess consumers' understanding of food safety. This section covered subjects such as microorganisms causing food poisoning, personal and food hygiene, high‐risk individuals, and proper cleaning practices. The answer option “don't know” was included to help prevent respondents from mistakenly selecting the correct answer and to reduce randomization bias. Each of the two “yes” answers received zero points, while each “yes” response was assigned one point. A respondent could attain a maximum score of 18 for food safety knowledge by correctly answering all questions. The frequency of purchases and reasons for choosing SVFs, along with the level of consumer trust in the safety of ready‐to‐eat SVFs, were discussed in the part focused on street food purchasing. The survey consisted of three sections. The initial section, “Street Food Purchasing,” gathered information on how often people buy SVF and their motivations, as well as their confidence in the safety of ready‐to‐eat SVFs. The second section, titled “Microbial Food Safety Hazard Awareness,” posed questions regarding types of food safety hazards, concerns about food safety or outbreaks of FBDs related to ready‐to‐eat SVFs, and the level of awareness and understanding of foodborne bacteria and related diseases. The third and final section focused on consumer perceptions of food safety risks associated with the buying and selling of ready‐to‐eat SVFs.

### 2.5 Validity and Reliability of the Questionnaire

The initial English version of the questionnaire utilized in this study was derived from previous research [4], with minor modifications. However, the questionnaire was translated into Pashto during the data collection phase, as it was carried out in regions where Pashto is spoken. A multilingual translator translated the questionnaire into two languages, and a different research assistant verified its accuracy. Additionally, another independent multilingual research team member conducted a back‐translation of the questionnaire to ensure consistency and avoid bias. Before the final version of the questionnaire was distributed, a pilot study involving 50 consumers of ready‐to‐eat SVFs was performed to ensure that all questions were understood. It is important to note that the outcomes of the piloted survey are not included in this study. The range of Cronbach's alpha values, from 0.710 to 0.884, indicates that the various sections of the questionnaire exhibited a satisfactory level of internal consistency [14].

### 2.6 Statistical Analysis

The data evaluation was conducted using the Statistical Package for the Social Sciences (SPSS) software (version 23.0). To summarize the variables of interest, descriptive statistics, including response frequencies/percentages, and a *t*‐test were used. A *p*‐value lower than 0.05 (indicating significance at a 95% confidence level) was considered statistically significant.

## 3 Result

The sociodemographic information of the respondents is presented in Table 1. A total of 500 individuals participated in the study; the majority were male (89.0%), while female food handlers represented 11.0%. Almost half of the respondents indicated they have completed at least an undergraduate degree. The age distribution reveals that a substantial proportion, 74.2%, of food handlers are aged between 18 and 25, whereas only 5.6% belong to the 36 to 45 age group. Less than half of the street food vendors (40.0%) reported earnings below 10,000 Afghani monthly, while 27.4% had monthly incomes between 10,000 and 15,000 Afghani. In this research, more than half of the street vendors were single at 64.8%, compared to 35.2% who were married (Table 1).

**Table 1:** Socio‐demographic characteristics of study participants (*N* = 500).

Variables	Categories	Frequency	Percentage (%)
Gender	Male	445	89.0
	Female	55	11.0
Age	18–25	371	74.2
	26–35	101	20.2
	36–45	28	5.6
Occupation	Unemployed	36	7.2
	Employed	123	24.6
	Student	301	60.2
	Business	14	2.8
	Others	26	5.2
Education level	No formal education	67	13.4
	Primary education	21	4.2
	Secondary	42	8.4
	Higher secondary	135	27.0
	Undergraduate	198	39.6
	Graduate or above	37	7.4
Household income	Below 10,000	200	40.0
	10,000–15,000	137	27.4
	15,001–20,000	45	9.0
	20,001–25,000	50	10.0
	25,001–30,000	58	11.6
	Above 30,000	10	2.0
Marital status	Single	324	64.8
	Married	176	35.2
	Divorced/separated	0	0.0
Residence	City	321	64.2
	Sub urban	84	16.8
	Rural	95	19.0

### 3.1 Socio‐Demographic Variables and Their Association With Food Safety Knowledge

Table A1 displays consumers' awareness regarding food safety in relation to SVFs. Less than half of the respondents acknowledged that *Salmonella* (43.8%), *Staphylococcus* (40.4%), and the hepatitis A virus (43.8%) are pathogens responsible for FBDs. The participants demonstrated a lack of sufficient understanding of foodborne pathogens. More than half (60.8%) of those surveyed identified the possibility of diarrhea transmission through food. A significant portion of the respondents agreed that wearing gloves while handling food (71.7%) and properly washing hands before food preparation (77.4%) can help lower the risk of contamination (see Table A1). The research indicated that the average knowledge score regarding food safety was 10.44 (with a standard deviation of 3.75, and scores ranging from 3 to 18) on an 18‐point scale. Furthermore, the overall accuracy rate for the knowledge assessment was 57.98% (calculated as 10.44/18 × 100), reflecting a moderate level of understanding.

### 3.2 Reasons for Buying and Consuming Food From Street Vendors and Their Conviction in Food Safety

**Figure 1:**
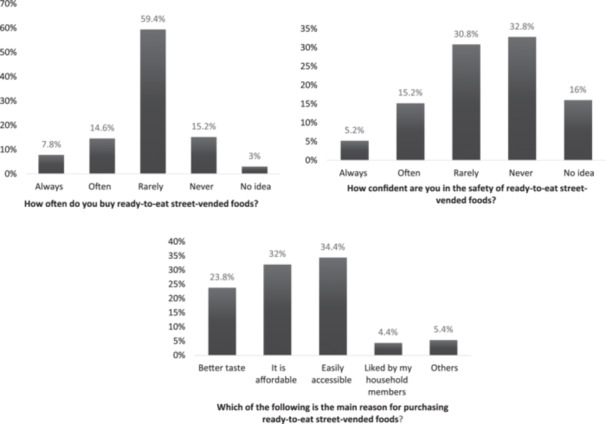
Consumers' responses regarding their confidence in and reasons for purchasing and consuming street‐vended foods (*N* = 500).

Less than one in four respondents (20.4%) indicated that they “always” or “often” have confidence in the safety of ready‐to‐eat SVFs. The most frequently cited reasons for purchasing and consuming ready‐to‐eat SVFs were ease of accessibility (34.4%), followed by affordability (32%) and superior taste (23.8%) (Figure 1). In terms of their confidence in the safety of SVFs, respondents; answers varied significantly (*p* < 0.05) based on subgroups such as age, occupation, education, and residence. Additionally, there was a notable difference (*p* < 0.05) in the reasons for purchasing ready‐to‐eat SVFs among different categories related to age, income, and health certification (Table 2).

**Table 2:** Consumers' responses regarding their confidence in and reasons for purchasing and consuming street‐vended foods within different groups.

Attitudes	Variables								
	Gender	Age	Occupation	Education	Income	Residence	Marital status	Food safety training	Health certificate
How often do you buy ready‐to‐eat street‐vended foods?	0.994	0.549	0.223	0.122	0.035	0.151	0.281	0.143	0.006
	(−4.8)	(−0.04)	(−2.052)	(−0.286)	(1.288)	(−0.532)	(0.233)	(−0.975)	(−1.753)
How confident are you in the safety of ready‐to‐eat street‐vended foods?	0.014	**0.01**	**0.003**	0.035	0.363	**0.003**	0.065	0.54	0.028
	(−2.98)	(−1.259)	(−2.144)	(−0.078)	(2.283)	(−0.949)	(0.312)	(−2.458)	(0.728)
Which of the following is the main reason for purchasing ready‐to‐eat street‐vended foods?	0.798	0.661	0.231	0.232	0.86	0.623	0.433	0.145	0.168
	(−5.184)	(−3.093)	(−2.791)	−0.732	(−0.314)	(−5.398)	(−0.786)	(0.168)	(2.4)

*Note:* Data are presented here as *p*‐value (t/F). Bold values indicate statistical significance at *p* < 0.05. *p*‐value was determined by an independent sample *t*‐test.

### 3.3 Microbial Food Safety Hazards Awareness Among Street‐Vended Food Consumers

More than half of the participants indicated that they were uncertain about the bacterial (62.0%), fungal (10.2%), and other food safety risks (27.8%) associated with ready‐to‐eat SVFs. Almost a quarter of the participants (18.2%) reported that they were “always” concerned, and one‐third (31.2%) expressed worries about FBDs when purchasing ready‐to‐eat SVFs. Approximately 23.0% of the participants were “very aware,” and around half (49.8%) were “aware” that specific foodborne bacteria can lead to diseases that may result in death, while 16.4% were not aware of this. *E. coli* and *Salmonella* were recognized as the most familiar, whereas *Campylobacter jejuni* and *Listeria monocytogenes* were cited as the least recognized foodborne pathogens by the participants (Table A2).

### 3.4 Attitude of Street‐Vended Food Consumers on the Sale and Purchase of Street‐Vended Food

**Figure 2:**
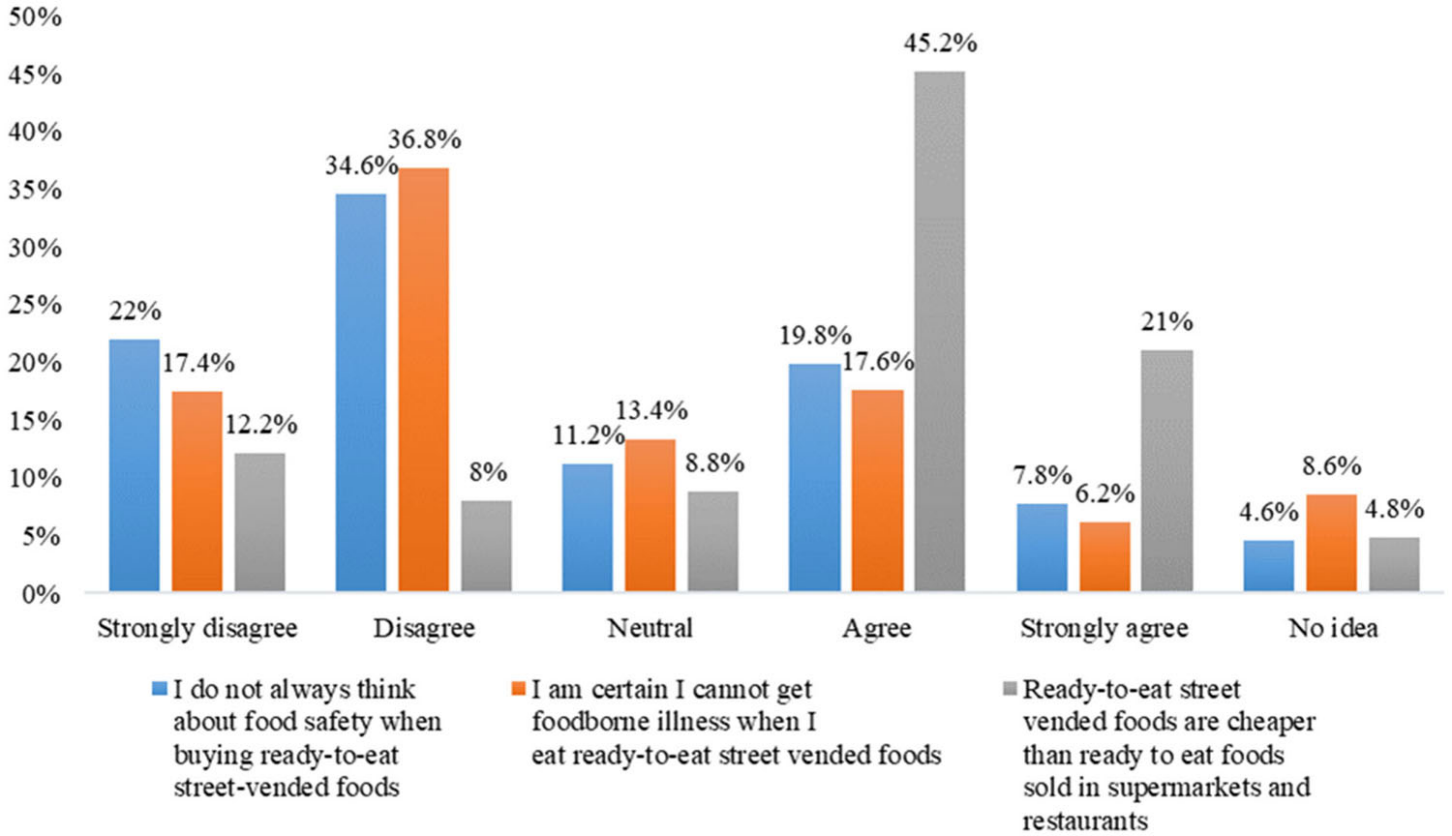
Attitude of street‐vended food consumers on the sale and purchase of ready‐to‐eat street‐vended food (*N* = 500).

Almost one‐third of the survey participants (27.6%) either strongly agreed (7.8%) or agreed (19.8%) with the statement “I do not always consider food safety when purchasing ready‐to‐eat SVFs,” in contrast to 22% who either strongly disagreed or disagreed (34.6%). In a similar vein, two‐thirds of the participants (66.2%) either strongly agreed (21%) or agreed (45.2%) with the statement “Ready‐to‐eat SVFs are less expensive than ready‐to‐eat meals available in supermarkets and restaurants” (Figure 2). Responses from different subgroups categorized by age, education, residence, and income did not show significant differences (*p* < 0.05) regarding their thoughts on food safety while buying ready‐to‐eat SVFs or their belief that ready‐to‐eat SVFs were more affordable than those offered in supermarkets and restaurants (Table 3).

**Table 3:** Attitude of street‐vended food consumers on the sale and purchase of ready‐to‐eat street‐vended food within different groups (*N* = 500).

Attitudes	Variables								
	Gender	Age	Occupation	Education	Income	Residence	Marital status	Food safety training	Health certificate
I do not always think about food safety when buying ready‐to‐eat street‐vended foods	0.008	0.023	0.116	0.472	0.015	0.042	0.054	0.307	0.385
	(1.576)	(0.933)	(−3.036)	(−0.017)	(0.138)	(1.976)	(2.16)	(−1.77)	(0.434)
I am certain I cannot get foodborne illness when I eat ready‐to‐eat street‐vended foods	0.009	**0.000**	0.047	0.728	**0.000**	0.065	0.906	0.835	0.694
	(3.308)	(2.073)	(0.224)	(−1.698)	(1.456)	(1.893)	(−1.559)	(−0.763)	(−1.438)
Ready‐to‐eat street‐vended foods are cheaper than ready‐to‐eat foods sold in supermarkets and restaurants	0.022	0.399	**0.000**	0.814	0.336	0.08	0.006	0.012	0.11
	(−0.842)	(1.495)	(−1.651)	(−0.093)	(−0.553)	(0.055)	(1.389)	(−2.262)	(−2.015)

*Note:* Data are presented here as *p*‐value (t/F). Bold values indicate statistical significance at *p* < 0.05. *p*‐value was determined by an independent sample t‐test.

**Table A1:** Assessment of food safety knowledge of street food consumers in Kandahar City (*N* = 500).

Statements	Number of responses (%)					
	Correct		Wrong		Don't know	
1. Abortion in pregnant women can be induced by a food‐borne disease	220	(47.5)	74	(16.2)	206	(6.2)
2. Bloody diarrhea can be transmitted by food	323	(60.8)	52	(11.3)	125	(27.90
3. Swollen cans can contain microorganisms	254	(50.9)	54	(10.9)	192	(38.)
4. During an infectious disease of the skin, it is necessary to take leave from work	312	(67.2)	71	(10.9)	117	(21.9)
5. Eating and drinking in the workplace increases the risk of food contamination	356	(72.1)	74	(11.7)	70	(16.2)
6. Hepatitis A virus is a food‐borne pathogen	207	(43.8)	80	(14.3)	213	(41.9)
7. Microbes are in the skin, nose, and mouth of healthy handlers	289	(59.6)	103	(18.5)	108	(21.9)
8. Salmonella is among the food‐borne pathogens	210	(43.8)	63	(15.1)	227	(41.1)
9. Staphylococcus is among the food‐borne pathogens	189	(40.4)	67	(13.2)	244	(46.4)
10. Typhoid fever can be transmitted by food	201	(37)	101	(22.3)	198	(40.8)
11. Using gloves while handling food reduces the risk of food contamination	358	(71.7)	64	(12.1)	78	(16.2)
12. Washing hands before work reduces the risk of food contamination	383	(77.4)	53	(10.6)	64	(12.1)
13. AIDS can be transmitted by food	204	(42.3)	154	(29.1)	142	(28.7)
14. Children, healthy adults, pregnant women, and older individuals are at equal risk for food poisoning	286	(58.9)	78	(18.1)	136	(23)
15. Food prepared in advance reduces the risk of food contamination	277	(57)	121	(19.6)	102	(23.4)
16. Proper cleaning and sanitization of utensils decreases the risk of food contamination	384	(78.9)	43	(8.3)	73	(12.8)
17. Reheating cooked foods can contribute to food contamination	286	(58.1)	128	(21.9)	86	(20)
18. Washing utensils with detergent leaves them free of contamination	290	(76.6)	38	(8.68)	172	(14.7)

*Note:* Each “yes” and “no” option for each statement is considered a correct and incorrect response, respectively.

**Table A2:** Microbial food safety hazards awareness of street‐vended food consumers in Kandahar City (*N* = 500).

Q 1: How concerned are you about the safety of street‐vended foods in terms of contamination with the following?		
Types of hazard	Frequency of concern about hazards; *n* (%)	
	Number of responses	
Bacteria	310	(62.0)
Fungi	51	(10.2)
Other hazards: Chemical, Lead, Mercury, and Aluminum.	139	(27.8)

Q 2: How often do you get worried about food‐borne diseases when you buy ready‐to‐eat foods from any of the following outlets?										
Types of retail outlets	Frequency of concern about hazards; *n* (%)									
	Uncertain		Never		Rarely		Often		Always	
Supermarket	152	(30.4)	113	(22.6)	133	(26.6)	67	(13.4)	35	(7.0)
Street food market	77	(15.4)	97	(19.4)	127	(25.4)	125	(25.0)	74	(14.8)
Restaurant	118	(23.6)	85	(17.0)	175	(35.0)	79	(15.8)	43	(8.6)
Shebeen	71	(14.2)	75	(15.0)	107	(21.4)	156	(31.2)	91	(18.2)

Q 3: Which of the following, in your opinion, is the most important food safety issue of concern nowadays?		
Food safety concern	Number of responses	Percentage
Presence of allergens	90	(18.0)
Foodborne diseases caused by bacteria	147	(29.4)
Sale of expired food	119	(23.8)
Unknown food sources	73	(14.6)
Too many junk foods	18	(3.6)
No idea	53	(10.6)

Q 4: Are you aware that certain foodborne bacteria can cause diseases that may lead to death?		
Percentage	Number of responses	Percentage
Very aware	115	(23.0)
Aware	249	(49.8)
Not aware	82	(16.4)
No idea	54	(10.8)

Q 5: Have you ever heard about any of the following foodborne bacteria?				
Foodborne bacteria	Yes; *n* (%)		No; *n* (%)	
*Escherichia coli*	86	(17.2)	69	(13.8)
*Salmonella*	75	(15)	78	(15.6)
*Campylobacter jejuni*	68	(13.6)	44	(8.8)
*Listeria monocytogenes*	40	(8)	40	(8)

## 4 Discussion

The present study elucidates the understanding and awareness of consumers of SVFs regarding food safety, FBDs, and microbial hazards. Our research findings indicate that consumers of SVFs in Kandahar City have a moderate understanding (57.98%) of food safety. This moderate knowledge of food safety is accompanied by participants' high awareness of hand hygiene practices (77.4%) and the use of gloves during food handling (71.7%), both of which are crucial practices that reduce the risk of disease transmission and food contamination. These findings align with earlier studies conducted in Saudi Arabia [15], Bangladesh [4], Malaysia [16], Johannesburg, South Africa [17], and Haiti [18], which reported that street food consumers displayed a moderate understanding of food safety. One study reported that street food vendors have a moderate level of knowledge but demonstrate negative attitudes and poor practices regarding food safety [19]. Nevertheless, the moderate understanding of food safety among SVF consumers highlights an urgent need to bolster health promotion and educational efforts targeted at this group. Furthermore, our research indicated that knowledge levels varied across different socio‐demographic factors (such as gender, age, and educational background). In line with findings from prior studies [4, 15, 20], our results reveal that females had a better grasp of food safety compared to males. The increased awareness among females regarding food safety may be linked to societal norms that assign women the role of primary food preparers in households, thus giving them deeper insights into food safety compared to men, who often participate less in household food preparation due to existing patriarchal norms. One study suggests that this pattern could be the result of female vendors being perceived as more dependable, safer, cleaner, friendlier, and more patient with food customers [21].

Additional important demographic factors revealed by our research included age and educational attainment. Consumers in the older age range (36–45 years) exhibited a better comprehension of food safety compared to their younger peers (18–25 years). This finding is consistent with previous literature [8], which indicated that older individuals generally have a greater awareness of food safety. One possible explanation for this trend could be that older consumers have gained significant experience with food contamination and related concerns, leading to a superior understanding of food safety. On the other hand, studies have shown that younger consumers may often display higher levels of food safety knowledge, as they tend to be more receptive to various sources of information (like media), which are essential for spreading awareness about food safety. Additionally, respondents who had educational qualifications beyond secondary school indicated a greater level of knowledge regarding food safety, as opposed to those without formal education or with only secondary schooling. This pattern has been supported by earlier research involving food handlers and consumers in different contexts [22, 23]. One study found that the food safety attitudes of the youngest consumers were notably better than those of older age groups [21].

Additionally, consumers who buy safe food generally possess greater knowledge and are more inclined to pay premium prices [24]. Altogether, our results highlight the importance of enhancing the understanding and awareness of Kandahar City's consumers regarding SVFs through food safety education and interventions (including food safety training, awareness campaigns, etc.), placing particular emphasis on young adult males and those with lower educational levels, to reduce FBDs and microbial risks linked to the consumption of SVFs.

Our investigation revealed that the primary factors affecting the consumption of SVFs in Kandahar City were ease of access and economic affordability. Our results are consistent with previous research regarding affordability but diverge in terms of pricing [25, 26]. Other influencing factors include individual tastes, such as a preference for flavor and the convenience of access. This finding supports previous studies [27]. The consumption of SVFs is particularly prevalent among consumers facing economic challenges in Kandahar City, most of whom are either unemployed or have low incomes, which explains the emphasis on easy accessibility for procuring and consuming SVFs.

With respect to the understanding of microbial food safety risks, our research revealed that although consumers are aware of foodborne bacteria, their knowledge of particular pathogens (e.g., *E. coli*, *Salmonella*, *Campylobacter jejuni*, and *Listeria monocytogenes*) linked to FBDs remains insufficient. This finding is supported by additional studies from Bangladesh [28, 29], China [21], Vietnam [18], and Haiti [30], where more than 90% of vendors were unaware of these pathogens. The implications of this result indicate that while consumers are not completely uninformed about food safety issues, the difficulty in identifying specific food pathogens might explain the low awareness levels regarding microbial food safety hazards. *E. coli* and *Salmonella* were identified as the most commonly recognized food pathogens in this research, aligning with earlier studies conducted among adult SVF consumers in a Johannesburg municipality [7].

Interestingly, around 76% of the participants revealed uncertainty about food safety issues, such as microbial contamination, chemical risks, and expiration dates, while they also recognized the danger of contracting FBDs by consuming SVFs. This finding contrasts with a prior study that indicated over three‐fourths (79.7%) of food vendors were knowledgeable about food safety and hygiene practices, and another studies [20], and [31] noted that a significant majority (76%) of street food vendors had a poor understanding of food safety, with only 14% of vending locations meeting sanitary standards. This suggests that consumers of SVFs have a reduced awareness of food safety and related health risks, despite acknowledging potential dangers. Our results showed that concerns regarding the safety of ready‐to‐eat SVFs were not significantly different from those related to ready‐to‐eat options from restaurants or supermarkets. SVF consumers likely perceive the advantages (such as affordability and convenience) to outweigh any risks involved in consuming SVFs. Research findings indicate that many consumers choose street foods for their moderate pricing compared to formal dining establishments, often overlooking the unsanitary conditions at the vending locations [26]. Another study found that a significant portion (62%) of respondents considered the cost of food to be a crucial factor when deciding to purchase SVFs [25].

The knowledge consumers possess about the food safety of SVFs may have a positive effect on their attitudes, which could lead to better food safety behaviors and practices [16]. Nevertheless, earlier studies that have explored consumers' food safety concerns have found that, despite their worries about food safety, consumers often partake in improper practices that could lead to FBDs [16, 32]. Using media platforms (like television, newspapers, and radio) could aid in changing behaviors that help reduce the risk of FBDs. Therefore, it is recommended to conduct additional research on food safety among SVF consumers using knowledge, attitudes, and practices (KAP) modeling to improve understanding of how consumer knowledge can be converted into practical behavior.

The safety of street‐vendor foods can be improved through health promotion and awareness efforts that involve various stakeholders, such as local government agencies, street food vendors, policymakers, consumer advocacy organizations, standard‐setting bodies, and different non‐governmental organizations. Additionally, public health authorities need to engage in coordinated actions to enhance the safety of street foods and improve the knowledge and practices of street food vendors through targeted informational campaigns, along with strict oversight and regulation of these vendors. These actions will maintain food safety standards, ensuring that even when consumers show indifference towards food safety, the risks tied to foodborne and microbial hazards are reduced. Moreover, it is crucial to motivate consumers to focus on proper food safety practices to support the efforts made by street food vendors.

## 5 Strengths and Limitations

This investigation has several significant strengths. It is one of the first studies focused on understanding the awareness and knowledge of consumers in Kandahar City regarding food safety, FBDs, and microbial risks associated with SVFs. As a result, the outcomes add to the growing body of scholarly literature and provide insights into awareness levels, knowledge, and factors affecting food safety and microbial risks related to ready‐to‐eat SVFs in developing countries. A key strength of our analysis lies in its methodological rigor and thorough analytical frameworks. Cross‐sectional studies effectively highlight specific areas where SVF consumers lack knowledge, particularly regarding foodborne pathogens and safe food handling practices. They allow for the evaluation of current food safety practices among consumers of SVFs, presenting a temporal snapshot of adherence to safety guidelines. The findings could be used to develop targeted educational programs and training efforts to improve food safety practices. Additionally, this research is one of the initial studies exploring the factors that influence food safety knowledge and practices among SVF consumers in a region where FBDs frequently occur each year. Our results could provide essential data for future interventions aimed at improving food safety knowledge and practices.

However, this study has certain limitations that should be considered when interpreting the findings. The design is cross‐sectional, capturing data at a single point in time, which limits the ability to observe changes in knowledge or practices over time. Self‐reported data might introduce biases, as participants could overstate their knowledge or adherence to safety measures. For example, food safety practices were assessed through self‐reported information rather than through direct observation, which could lead to potential reporting bias. Nevertheless, we used a 5‐point Likert scale to evaluate food safety practices, which may help reduce the likelihood of reporting bias. The results might not apply to other regions or populations due to cultural and environmental differences in food handling practices. For instance, considering the specific municipal areas in Kandahar City analyzed in this study (i.e., 15 municipal areas), the findings might not accurately represent the entire province or region. Moreover, this study did not include an evaluation of the microbiological quality of SVFs, which needs further research to strengthen the scientific integrity of this KAP study. While cross‐sectional studies provide valuable insights into food safety among SVF consumers, they should be complemented with longitudinal studies to monitor changes and improvements over time.

## 6 Conclusion

To sum up, the cross‐sectional study conducted to evaluate knowledge and awareness about food safety, FBDs, and microbial risks among consumers of SVFs in Kandahar City, Afghanistan, reveals that the primary demographic of respondents was male. Almost half of the participants reported having achieved an undergraduate degree or higher educational credentials. The participants' understanding of foodborne pathogens was considerably lacking. A significant majority of respondents acknowledged that wearing gloves while handling food and washing hands before food preparation can reduce the risk of contamination. More than half of the respondents expressed uncertainty about bacterial contamination, while awareness of fungal and other food safety risks related to ready‐to‐eat SVFs was low. *E. coli* and *Salmonella* were recognized as the most common foodborne pathogens, whereas *Campylobacter jejuni* and *Listeria monocytogenes* were among the least known pathogens by the respondents. The results drawn from our study suggest that consumers of SVFs in Kandahar City have a moderate level of knowledge regarding food safety. When analyzing by gender, females exhibited a greater understanding of food safety than their male counterparts. There is a need for concerted efforts to promote the implementation of Hazard Analysis Critical Control Point (HACCP) standards among all tiers of street‐vended food handlers to ensure compliance with food safety regulations. Additionally, our findings carry important implications for Kandahar City's ability to achieve Sustainable Development Goal (SDG) 2.2 by 2030. Ultimately, there is an immediate need for intervention strategies and longitudinal research involving a wide range of microbiological samples from SVFs in Kandahar City to clarify the connections between the consumption of such foods and disease patterns, thereby aiding in the decrease of FBDs in Kandahar City.

## Author Contributions

**Ahmadullah Zahir:** writing – review and editing, writing – original draft, visualization, validation, conceptualization. **Safiullah Jauhar:** writing – review and editing, conceptualization. **Sayeed Hikmatullah Anis:** conceptualization and validation, methodology, formal analysis.

## Ethics Statement

Ethical approval for this study was obtained from the Ethical Review Committee of the Afghanistan National Agricultural Sciences & Technology University, Kandahar 3801, Afghanistan, with REF. No. ANASTU/VET/EC/1403/12.

## Consent

All authors have read and approved the final version of the manuscript. Ahmadullah Zahir had full access to all of the data in this study and takes complete responsibility for the integrity of the data and the accuracy of the data analysis.

## Conflicts of Interest

The authors declare no conflicts of interest.

## Transparency Statement

The lead author Ahmadullah Zahir affirms that this manuscript is an honest, accurate, and transparent account of the study being reported; that no important aspects of the study have been omitted; and that any discrepancies from the study as planned (and, if relevant, registered) have been explained.

## Data Availability

The data that support the findings of this study are available from the corresponding author upon reasonable request.
